# Role of Second-Look Ureterorenoscopy After Endoscopic Treatment of Upper Tract Urothelial Carcinoma

**DOI:** 10.3390/cancers18172893

**Published:** 2026-09-07

**Authors:** Mohammad Abufaraj, Beat Foerster, Tim Muilwijk, Gautier Marcq, Thomas Seisen, Roger Li, Leonardo L. Monteiro, Marco Bandini, Donald Schweitzer, Alexander Kenigsberg, David D’Andrea, Kees Hendricksen, Francesco Soria, Giuseppe Fallara, Steven Joniau, Evanguelos Xylinas, Marco Moschini, Morgan Rouprêt, Alberto Briganti, Philippe E. Spiess, Wassim Kassouf, Georgi Guruli, Hubert John, Dmitry Enikeev, Mounsif Azizi, Pierre Colin, Shahrokh F. Shariat

**Affiliations:** 1 Division of Urology, Department of Special Surgery, Jordan University Hospital, The University of Jordan, Amman 11942, Jordan; dr.abufaraj@gmail.com; 2 Department of Urology, Comprehensive Cancer Center Vienna, Medical University of Vienna, 1090 Vienna, Austria; 3 Department of Urology, Kantonsspital Winterthur, 8400 Winterthur, Switzerland; 4 Department of Urology, University Hospitals Leuven, 3000 Leuven, Belgium; 5 Division of Urology, Department of Surgery, McGill University Health Center, Montreal, QC H4A 0B1, Canada; 6 Department of Urology, Sorbonne University, GRC n°5, ONCOTYPE-URO, AP-HP, Urology, Hôpital Pitié-Salpêtrière, F-75013 Paris, France; 7 Department of Genitourinary Oncology, Moffitt Cancer Center, Tampa, FL 33612, USA; 8 Department of Urology, Urological Research Institute, Vita-Salute University, San Raffaele Scientific Institute, 20132 Milan, Italy; 9 Department of Urology, The Netherlands Cancer Institute-Antoni Van Leeuwenhoek Hospital, 1006 Amsterdam, The Netherlands; 10 Division of Urology, Virginia Commonwealth University, Richmond, VA 23284, USA; 11 Division of Urology, Department of Surgical Sciences, University of Studies of Torino, 10124 Turin, Italy; 12 Department of Urology, Bichat-Claude Bernard Hospital, Assistance Publique-Hôpitaux de Paris, Paris Descartes University, 75018 Paris, France; 13 Klinik für Urologie, Luzerner Kantonsspital, 6004 Lucerne, Switzerland; 14 Institute for Urology and Reproductive Health, I.M. Sechenov First Moscow State Medical University, 119991 Moscow, Russia; 15 Department of Surgery, Division of Urology, Centre Hospitalier de L’Université de Montréal (CHUM), Montreal, QC H2X 3E4, Canada; 16 Department of Urology, Hôpital Privé de La Louvière, Générale de Santé, 59042 Lille, France; 17 Department of Urology, University of Texas Southwestern Medical Center, Dallas, TX 75235, USA; 18 Department of Urology, Weill Cornell Medical College, New York, NY 10065, USA; 19 Karl Landsteiner Institute of Urology and Andrology, 1090 Vienna, Austria; 20 Department of Urology, Second Faculty of Medicine, Charles University, 150 06 Prague, Czech Republic

**Keywords:** upper tract urothelial carcinoma, kidney sparing surgery, ureterorenoscopy, second look surgery, local neoplasm recurrence

## Abstract

Objectives: To investigate the rate and predictors of recurrence at second-look ureterorenoscopy (URS) and to assess the adequate timeframe for second-look URS in upper tract urothelial carcinoma (UTUC). Methods: This multicenter retrospective study included 179 patients who underwent endoscopic kidney-sparing surgery (KSS) and second-look URS for non-invasive UTUC between 2004 and 2017. We performed logistic and Cox proportional hazard regression analyses to investigate the association between clinical parameters and second-look recurrence, recurrence-free survival (RFS), and cancer-specific mortality. The optimal duration to second-look URS was extrapolated using restricted cubic splines. Results: Overall, 66 (36.9%) patients experienced recurrence at second-look URS. After second-look URS, further recurrence was observed in 87 (48.6%) patients during a median follow-up of 12 months. Female gender (OR [odds ratio] 2.3, 95% CI [confidence interval] 1.1–4.7, *p* = 0.026) and tumor size > 1 cm (OR 3.3, 95% CI 1.2–9.5, *p* = 0.027) were independently associated with recurrence at second look. Second-look recurrence was a strong prognostic factor for RFS (HR 5.0, 95% CI 3.1–8.0, *p* < 0.001). Second-look URS between 5 and 12 weeks after initial endoscopic laser ablation was an independent favorable factor for RFS (HR 0.5, 95% CI 0.3–0.9, *p* = 0.014). Conclusions: Early recurrence at second-look URS appears to be one of the strongest predictive factors for RFS in patients undergoing endoscopic KSS. Our results suggest that second-look URS performed within 5 to 12 weeks is associated with a lower probability of further disease recurrence. Given that this interval was derived and tested in the same cohort, this finding should be regarded as hypothesis-generating and requires external validation before being adopted as a clinical standard.

## Simple Summary

The authors evaluated factors associated with tumor recurrence after laser treatment in patients with upper urinary tract cancer and examined the optimal timing of second-look ureteroscopy after the initial treatment. The authors found that the presence of a tumor at second-look ureteroscopy, tumor size greater than 1 cm, and female sex were associated with a higher risk of subsequent recurrence. The authors found that performing a second-look scope between five and twelve weeks after treatment appears to be the most appropriate timing to decrease the risk of tumor recurrence.

## 1. Introduction

Urothelial carcinoma is the fifth most frequent malignancy in Western countries [1,2]. Urothelial carcinoma of the upper urinary tract (UTUC) represents only 5–10% of urothelial malignancies [2,3]. Approximately 40% of UTUCs are non-invasive at diagnosis as compared with 75–85% of bladder urothelial carcinoma (UCB) [4].

Over the last decades, radical nephroureterectomy (RNU) was the standard treatment for non-metastatic UTUC patients. This paradigm has shifted, and kidney-sparing surgeries (KSS), such as segmental ureterectomy and ureterorenoscopy (URS) with laser ablation, were introduced as effective treatment options for specific non-invasive tumors [1,5]. Oncologic outcomes of KSS have shown comparable safety and efficacy in patients suffering from localized tumors with low-risk features [6].

The follow-up strategy after KSS is not clear yet. However, it is clear that a close and stringent follow-up is necessary because of the high risk of disease recurrence and progression [1,7]. The current guidelines recommend URS and urinary cytology at 3, 6 and 12 months after endoscopic KSS [1,5]. In contrast to follow-up cystoscopies after transurethral resections of the bladder, follow-up URS need to be performed under general anesthesia. Therefore, the morbidity of a stringent follow-up after KSS is considerable, and the optimal timing for follow-up URS needs to be assessed based on evidence.

Data showed that early recurrence can occur in more than half of the patients at the time of second-look URS, usually performed within 6 to 8 weeks [8]. These findings often led to change in disease management, specifically to RNU. Difficulty in staging at diagnosis, technical challenges and poor visibility during URS, and the lack of efficient adjuvant therapies advocate subsequent URS to ensure better cancer control and, theoretically, lower progression rates [1,9,10].

The primary aims of this study are to assess the rate of early recurrence and to suggest a timeframe for second-look URS. Secondary aims are to investigate predictors of early recurrence and prognostic factors for recurrence-free survival (RFS) and cancer-specific mortality (CSM).

## 2. Materials and Methods

### 2.1. Study Design

In this international retrospective analysis, we reviewed all UTUC patients who underwent endoscopic KSS and second-look URS within 16 weeks between 2004 and 2017 at ten academic centers in North America and Europe. We use the term ‘second-look URS’ to refer to the first planned endoscopic re-evaluation following initial endoscopic treatment, irrespective of the exact time interval. The absence of a standardized interval across participating centers is, in fact, the source of variation that this study uses to identify an optimal timeframe. The study protocol was approved by the institutional review boards at the leading site (no. 1566/2017) in addition to all cooperating centers; necessary data-sharing agreements were exchanged. Computerized databases with extracted data were merged, checked for inconsistencies, and frozen prior to analysis. This observational study was elaborated according to the STROBE statement for cohort studies [11].

### 2.2. Inclusion and Exclusion Criteria

**Figure A1:**
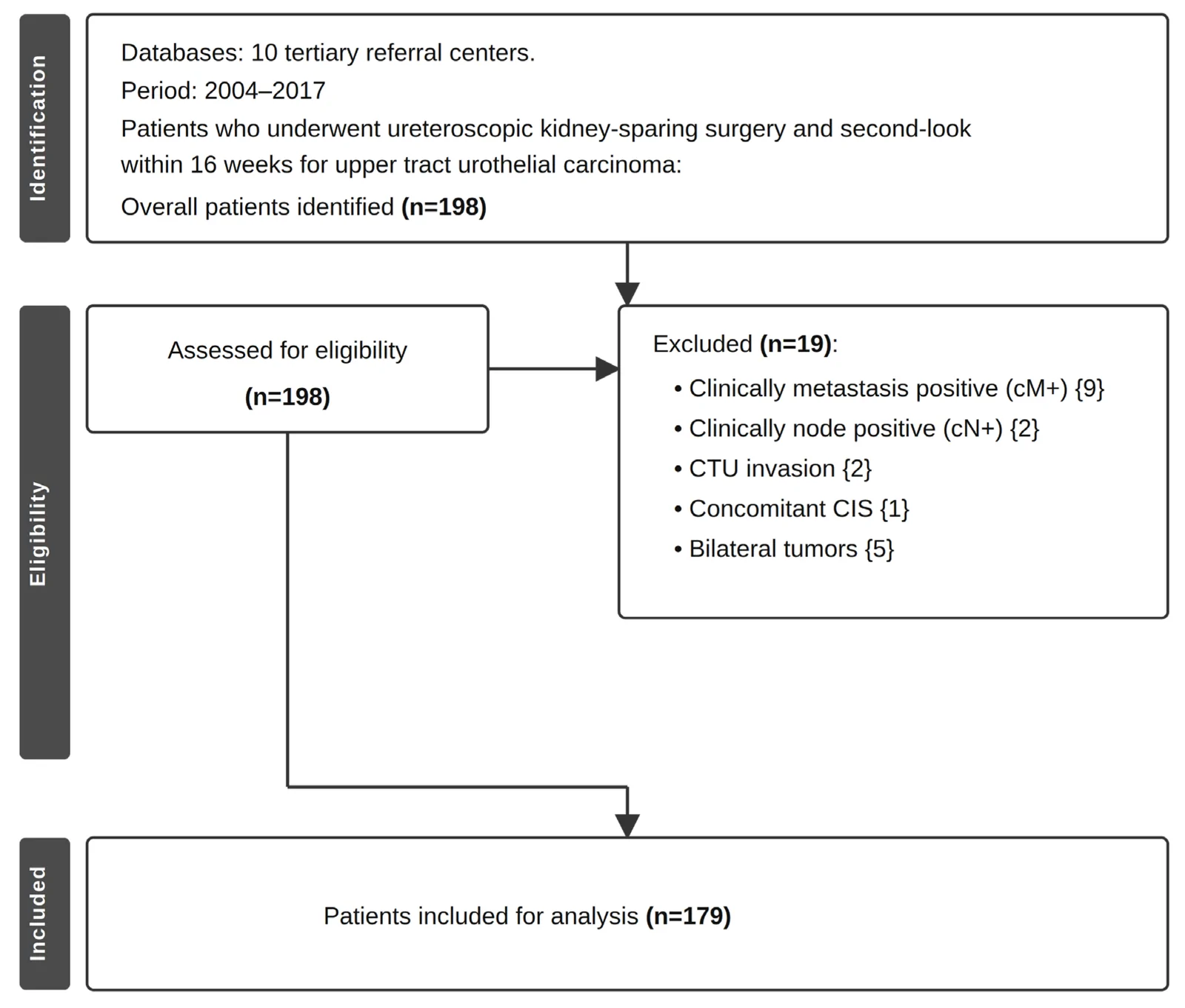
Flow diagram of patients’ selection process.

We included patients who received complete ureteroscopic laser ablation of UTUC with curative intent followed by a second-look URS within 16 weeks (Figure A1). Therefore, we excluded patients in palliative settings with evidence of lymph node metastasis (*n* = 2) [12], distant metastasis (*n* = 9) or Computed Tomography Urography (CTU) invasion (*n* = 2) [10]. We further excluded patients with concomitant carcinoma in situ (*n* = 1) and synchronous bilateral tumors (*n* = 5) to establish a homogeneous baseline cohort. As it is not completely clear yet which patients should be treated by endoscopic KSS, we did not exclude tumors with certain high-risk features, such as multifocality [4], high-grade differentiation, tumor size > 2 cm or hydronephrosis [10].

The presence of a lymph node or distant metastasis was a priori tested by cross-sectional imaging, and CTU invasion was defined as clinical stage ≥cT3, i.e., radiographic evidence of tumor extension beyond the ureteral or pelvicalyceal wall into the periureteric or peripelvic fat, with or without invasion of adjacent organs [10]. The presence of two or more tumors within the same upper urinary tract unit in cross-sectional imaging or visually during URS was considered multifocal. Genitourinary pathologists at each institution determined the tumor grading by the 2004 WHO/International Society of Urologic Pathologists (ISUP) consensus classification.

### 2.3. Follow-Up

The follow-up regimen across the participating institutions was not standardized, but all patients received a second-look URS within 16 weeks of the initial URS laser ablation. The selection of laser technology, such as Holmium:YAG or Thulium:YAG, and its settings for ablation and coagulation were determined by the operating surgeon. Generally, follow-up consisted of check-ups every 3–6 months for the first five years, and yearly thereafter. Examinations included medical history, physical examination, routine blood work, cystoscopy, and wash cytology with or without retrograde ureteropyelography of the upper urinary tract. CTU or magnetic resonance imaging was conducted every 6–12 months for the first five years or when clinically indicated. The frequency of follow-up URS was determined by the surgeon. The presence of recurrence was determined by visual inspection during URS and by retrieving biopsies from suspicious lesions. Recurrence was defined as tumor relapse in the upper urinary tract, within the previous resection area or other locations. We specified recurrence-free survival as the duration from second-look URS to subsequent tumor relapse during follow-up. New intravesical or urethral lesions were not considered as recurrence. The cause of death was determined by the attending physician, and confirmed by chart review and/or death certificates. For all patients with cancer-specific mortality, a previously recorded disease recurrence was mandatory.

### 2.4. Statistical Analysis

**Figure A2:**
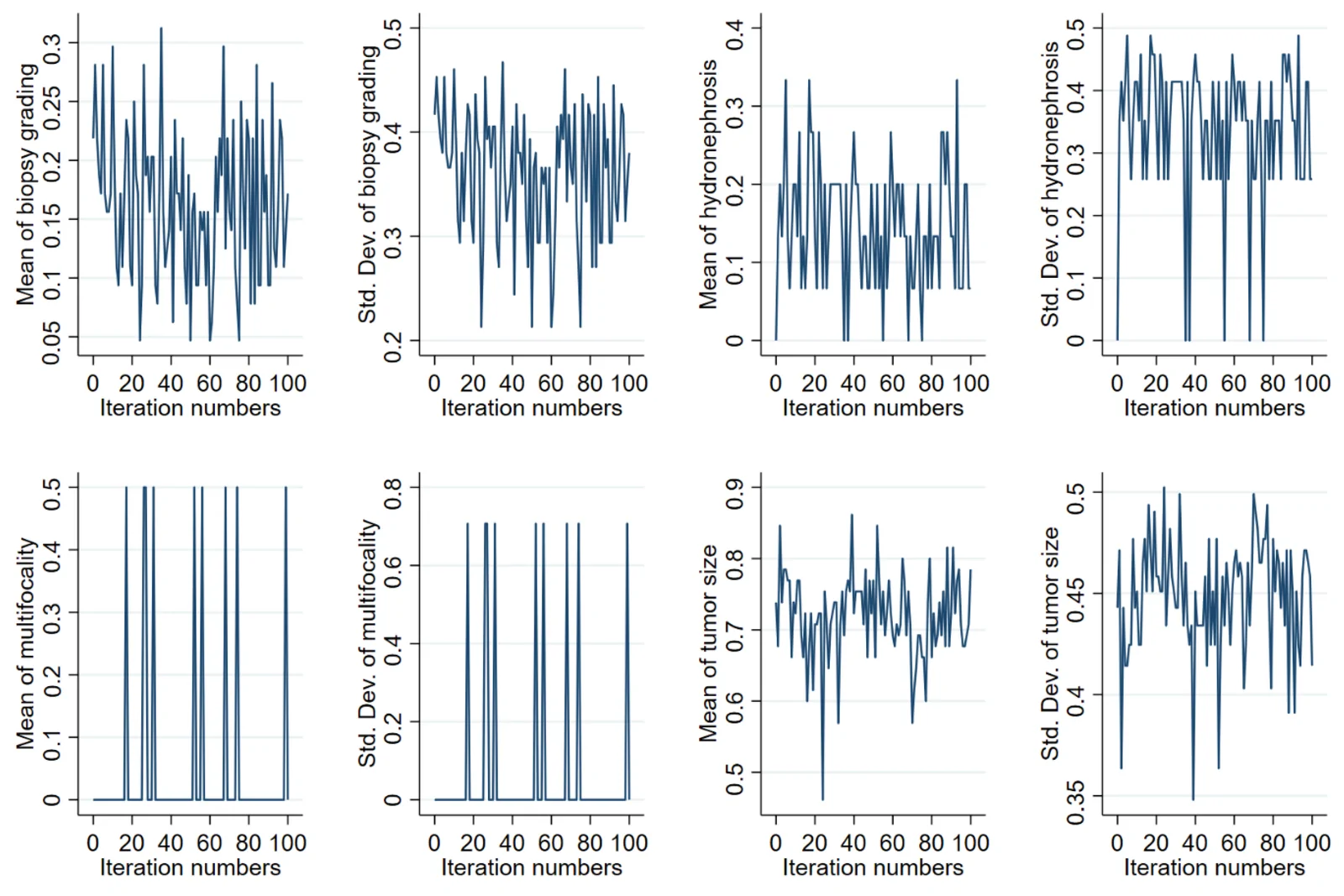
Trace plots summaries of imputed variables.

**Figure A3:**
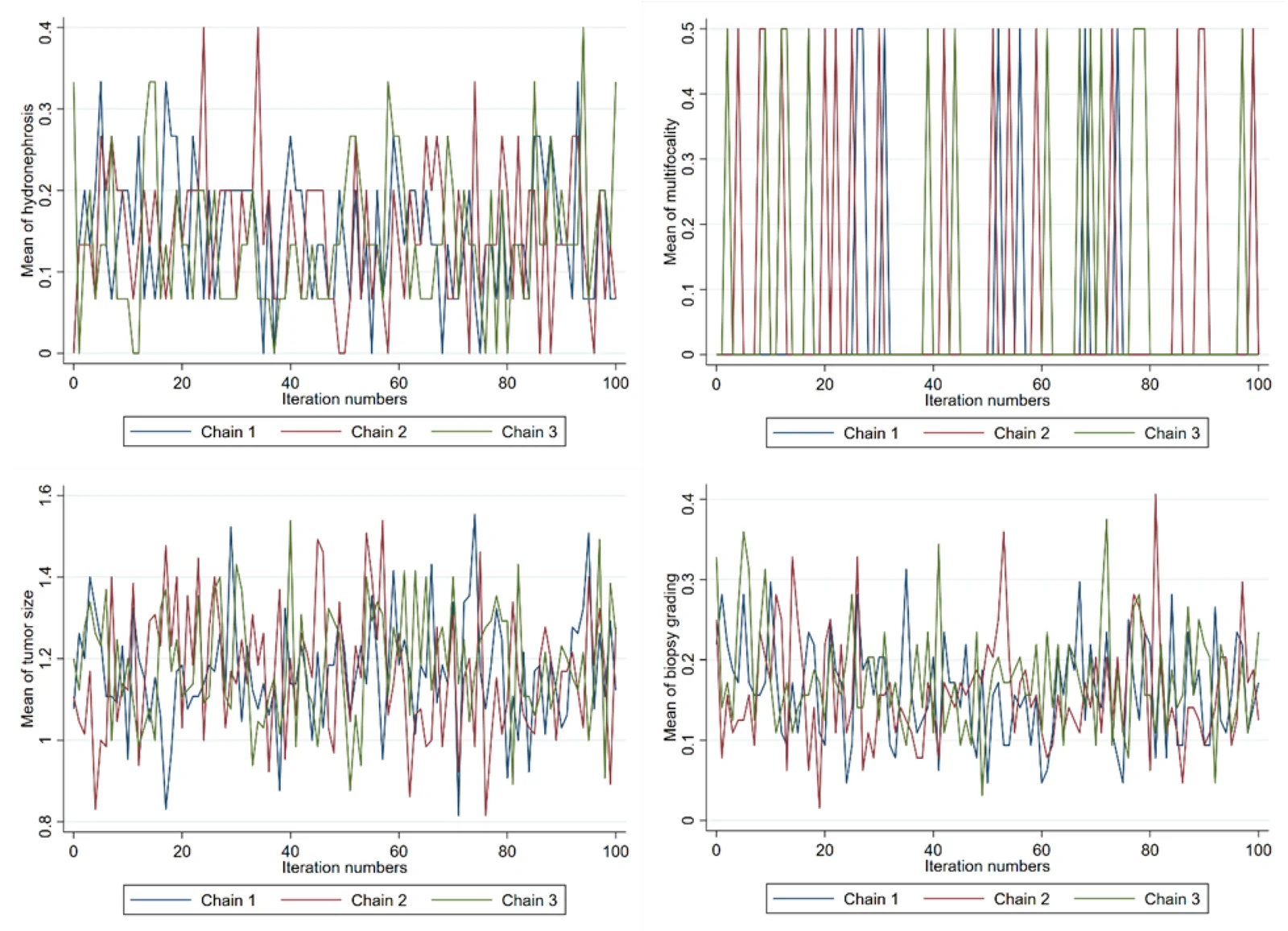
Trace plots summaries of imputed variables with three chains.

Pearson’s *X*^2^ and Mann–Whitney U tests were used to calculate the distributions of categorical and continuous clinical variables, respectively. We assumed all missing data to be missing at random (Table A1) and imputed their values using multiple imputation of chained equations [13]. We generated 20 imputed datasets to lessen the simulation error. Sequential logistic regression was performed for imputation of binary, multinominal logistic regression for nominal, ordered logistic regression for ordinal, linear regression for continuous, and predictive mean matching for the imputation of continuous categorical variables. Trace plots were computed to test for convergence and to identify an adequate number of iterations (Figure A2 and Figure A3) [14]. We utilized the augmented-regression approach to deal with perfect prediction of categorical variables [15].

We applied Rubin’s rules for all subsequent analyses to generate effect summaries across the imputed datasets. Logistic regression analysis was carried out to assess the predictive value of standard clinicopathologic and treatment-related factors on early recurrence during second-look URS. We performed Cox proportional hazard regression analyses to investigate the prognostic effect of previously mentioned parameters on RFS, and CSM. Time duration in weeks from initial URS treatment to second-look URS was added to the model with restricted cubic splines with knots at the tertiles to account for non-linearity [16]. The 5–12 week interval was derived by identifying the point of minimal predicted recurrence risk on the spline curve (8 weeks) and then extending to the upper boundary of its 95% confidence interval in both directions. The non-linearity of the spline model was tested against a linear-term-only model using a likelihood ratio test. The hazard ratio for the 5–12 week window was additionally assessed using bootstrap resampling (500 replications). The model was created using varying knot locations, and the location had little effect on the model results. We computed all statistical analyses using STATA 14.2 (Stata Corp., College Station, TX, USA). All tests were two-sided and *p*-values < 0.05 were considered statistically significant.

## 3. Results

### 3.1. Population Characteristics

We identified 179 patients with presumed non-metastatic non-invasive UTUC who underwent URS laser ablation and subsequently second-look URS within 16 weeks. The baseline clinicopathologic characteristics stratified by second-look recurrence are displayed in Table 1. Overall, 66 (36.9%) patients experienced early recurrence at second-look URS with a median duration to second-look of 9 weeks.

After second-look URS, recurrence was observed in 87 (48.6%) patients during a median follow-up of 12 months. Over a longer median follow-up of 27 months, 12 (6.7%) patients died of their disease. During follow-up, distant metastasis was documented in 35 patients (19.6%). Among the 66 patients with recurrence at second-look URS, 50 (75.8%) experienced subsequent recurrence during follow-up. Among the 113 patients who were free of recurrence at second-look, 37 (32.7%) subsequently developed recurrence.

### 3.2. Predictive Factors for Second-Look Recurrence

In multivariable logistic regression analyses (Table 2), female gender (OR [odds ratio] 2.3, 95% CI [confidence interval] 1.1–4.7, *p* = 0.026) and tumor size >1 cm (OR 3.3, 95% CI 1.2–9.5, *p* = 0.027) significantly increased the odds of recurrence at second-look URS, while high-grade biopsy during initial URS treatment did not predict recurrence at second-look URS (OR 1.1, 95% CI 0.4–2.9, *p* = 0.9). As a sensitivity analysis, we repeated the multivariable logistic regression restricted to patients with complete tumor size data (*n* = 114). Both tumor size >1 cm (OR 3.15, 95% CI 1.11–8.92, *p* = 0.031) and female gender (OR 2.46, 95% CI 1.01–6.00, *p* = 0.048) remained significant and similar in magnitude to the imputed-data estimates.

### 3.3. Prognostic Implications

**Figure 1:**
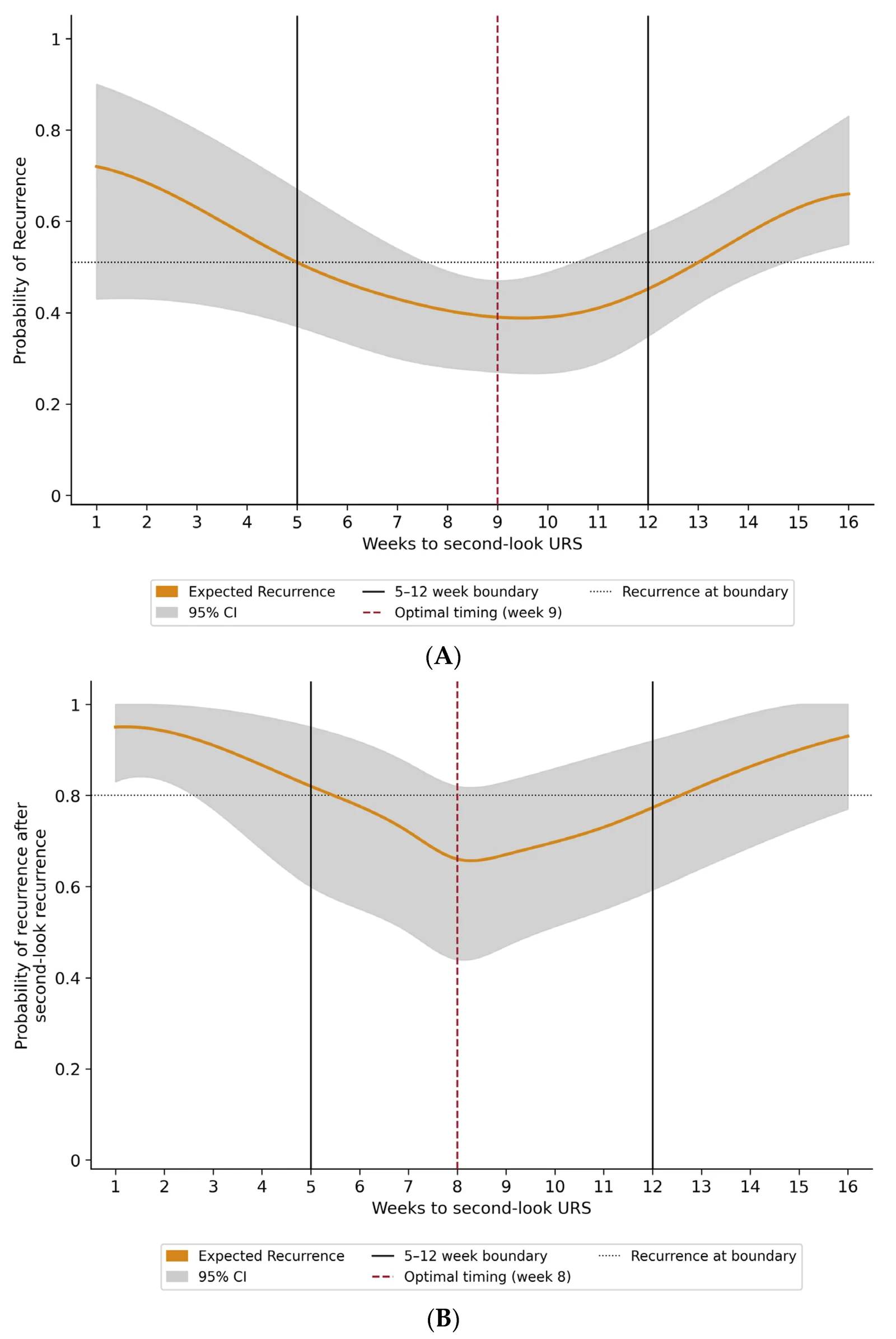
Restricted cubic spline models of the probability of upper tract recurrence as a function of time (weeks) from initial endoscopic laser ablation to second-look URS. (**A**) Probability of recurrence at second-look URS among all patients; (**B**) probability of further recurrence among patients who experienced recurrence at second-look URS. URS: ureterorenoscopy; CI: confidence interval.

**Figure 2:**
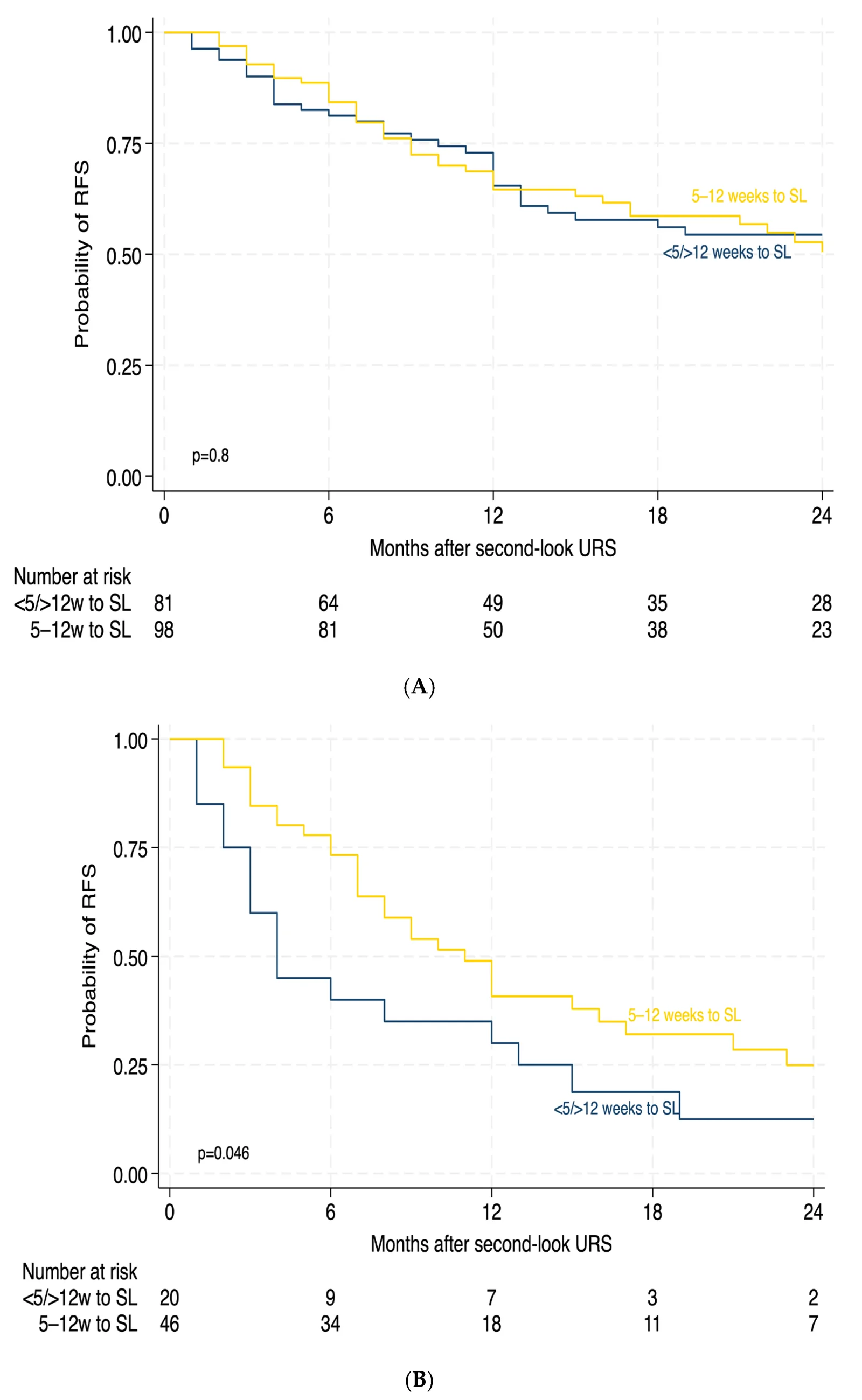
Kaplan–Meier estimates of recurrence-free survival (RFS) by time to second-look (SL) URS (5–12 weeks vs. <5/>12 weeks). (**A**) All patients (*p* = 0.8); (**B**) subgroup of patients with recurrence at second-look URS (*p* = 0.046). RFS: recurrence-free survival; SL: second look; w: weeks; URS: ureterorenoscopy; CI: confidence interval.

The risk of recurrence among all patients as a function of duration (weeks) from initial URS to second-look URS is depicted in Figure 1A. The lowest probability of recurrence (approximately 40%) can be observed at the 9-week mark, while in both directions along the time axis the probability increased. A likelihood ratio test comparing the restricted cubic spline model against a linear-term-only model confirmed significant non-linearity in the relationship between time to second-look URS and recurrence (LR chi^2^(1) = 43.62, *p* < 0.0001). On the subgroup of patients who experienced recurrence at second-look URS, the risk of recurrence as a function of time to second-look URS was lowest (approximately 65%) at the 8-week time point (Figure 1B). When defining the upper margin of the 95% CI level at the point of minimal risk (8 weeks) as the threshold, the optimal time duration from first URS treatment to second-look URS ranged between 5 and 12 weeks. Five weeks and 12 weeks are the lower and upper bounds of the 95% CI around the minimum-risk estimate (8 weeks); patients scheduled outside this range had a statistically higher predicted risk of recurrence. In Kaplan–Meier estimates for RFS, we did not observe significant difference (*p* = 0.8) between patients who had second-look within this range (5–12 weeks) and patients outside of this range (<5/>12 weeks) (Figure 2A). Conversely, a significant difference was noticed (*p* = 0.046) in the subgroup of patients with recurrence at the second-look (Figure 2B).

In multivariable Cox proportional hazard regression analysis, recurrence at second-look URS was independently associated with worse RFS (HR 5.0, 95% CI 3.1–8.0, *p* < 0.001) and 5–12 weeks duration to second-look URS with improved RFS (HR 0.5, 95% CI 0.3–0.9, *p* = 0.014), while tumor size and female gender did not reach significance in univariable analysis and were therefore not carried forward into the multivariable model (Table 3). A bootstrap analysis (500 replications) of the Cox model for the 5–12 week window yielded a hazard ratio of 0.54 (95% CI 0.29–0.99). In univariable analysis, second-look recurrence (HR 5.1, 95% CI 1.4–19.2, *p* = 0.016) and duration to second-look URS < 5 weeks (HR 6.6, 95% CI 1.1–40.3, *p* = 0.041) were significantly associated with CSM (Table 4). Multivariable analysis for CSM was not performed due to the overall low number of mortality events.

## 4. Discussion

In this collaborative effort, we found that female gender and tumor size >1 cm are independent predictors for early recurrence at second-look URS. Second-look recurrence is a strong adverse prognostic factor for RFS and CSM. Furthermore, we extrapolated the optimal time window from initial endoscopic KSS to second-look URS, which ranged between 5 and 12 weeks with a minimal probability of recurrence at approximately 8 weeks.

Female gender was found to be associated with increased risk of recurrence, Bacillus Calmette-Guérin immunotherapy failure, and cancer-specific and overall survival in patients with UCB [17,18,19,20]. In patients with UTUC a recent meta-analysis showed that female gender was not associated with increased risk of experiencing recurrence, progression or CSM after RNU [21]. The rate of intravesical recurrence following RNU, however, was lower in women [21]. Interestingly, we identified female gender as an independent predictor of recurrence at second-look URS. Such association was not observed for RFS after adjusting for the effect of second-look recurrence during further follow-up. Various gender-specific discrepancies have been discussed as underlying causes for worse prognosis in UCB, such as delayed diagnosis, hormonal and reproductive factors, and environmental and genetic factors, as well as thinner anatomic layers of the bladder [22,23,24,25]. Considering the relatively wide confidence interval (OR 2.3, 95% CI 1.1–4.7), subgroup size (48 women, 25 with second-look recurrence), and the unclear underlying biological explanation, the association between female sex and the outcome should be interpreted with caution. In UTUC, the latter cause is of concern given the risk of perforation during laser ablation. The location of the tumor also plays a role owing to technical and anatomical challenges [26]. Mid and proximal ureteral tumors, for example, are considered less favorable sites for endoscopic KSS [5]. In our analysis for early recurrence, we did not observe differences between distal, mid, proximal, and pelvic-calyceal tumors. Though, we identified tumor size (>1 cm) as a strong predictive factor for recurrence at second-look URS. Indeed, tumor extent is a major technical obstacle to complete endoscopic ablation. Nevertheless, the current guidelines advocate endoscopic KSS for tumors up to 1.5 or 2 cm if they harbor low-risk features due to their likelihood to remain non-invasive [1,27].

Previous studies that investigated the prognostic impact of tumor size and location in patients undergoing endoscopic KSS did not identify an association with progression-free survival [7,8]. These findings are in line with our findings knowing that recurrence at the second-look URS is one of the strongest prognostic factors in KSS treatment algorithms. Our results confirm the prognostic value of recurrence at second-look for both RFS and CSM, highlighting the importance of meticulous endoscopic laser ablation during the first intervention to avoid any residual disease. This is especially relevant because effective and practical adjuvant instillation therapies in the upper urinary tract are still lacking [9].

The currently available data identify high-grade disease as a prognostic factor for progression in UTUC patients treated with KSS [7]. In our analysis, we did not find an association between high-grade disease and worse RFS or CSM. These results appear to suggest that certain patients with a single high-risk feature, such as high-grade, could possibly benefit from an endoscopic approach. However, the low number of high-grade tumors limits statistical power, and since all patients were managed with curative-intent KSS, selection bias cannot be excluded, limiting generalizability to the broader high-grade UTUC population. Indeed, high-grade differentiation has already been demonstrated to be a strong adverse prognostic factor in UTUC patients treated with URS or RNU [7,28].

More than half of the patients experienced recurrence. We identified 5 to 12 weeks as the optimal time frame to perform second-look URS in low-risk UTUC patients with the lowest probability of recurrence during this interval. We further confirmed this association in a multivariable model adjusting for the effects of recurrence at second-look URS. Villa et al. suggested a second-look URS between 6 and 8 weeks following initial treatment, since within this time window [8]. However, conclusions from this study are limited owing to the lack of multivariable analysis assessing both disease recurrence and progression. Based on our results, we advocate an average duration to second-look URS of approximately eight weeks. Similar to UCB [29], the second look has profound prognostic implications.

Recent studies have further refined our understanding of second-look URS in KSS. A cancer detection rate of approximately 45% has been reported at second-look URS, with a positive finding independently associated with higher subsequent recurrence rates. On the other hand, low-grade, small tumors appear safe to defer beyond eight weeks. Importantly, second-look findings alone were not independently prognostic for recurrence or treatment conversion, and clinically significant events continue to occur beyond the current six-month surveillance window, arguing for extended follow-up [30,31].

From a survival perspective and using propensity-score adjustment, Gallioli et al. reported that patients undergoing second-look URS had significantly longer overall survival, with the benefit driven by other-cause rather than cancer-specific mortality [32]. This validates patient selection for conservative management rather than reflecting a direct antitumor effect of the procedure. Moreover, among tumors ≤10 mm, nine-month recurrence rates were remarkably low, supporting surveillance de-intensification in this subgroup.

This international retrospective study is not without limitations. The cohort is indeed well-selected, but the indication for endoscopic KSS was not standardized. All underwent KSS with curative intent, providing a homogeneous group of patients eligible for this treatment modality. Additionally, the treatment settings and devices were not standardized, as were the durations to second-look URS. Furthermore, this cohort predates newer laser technologies and more refined selection criteria, which limits the applicability of these findings to the current practice. Differences in follow-up protocols between institutions may also have affected recurrence detection rates, potentially introducing ascertainment bias. On the other hand, this heterogeneity opened up the possibility to investigate the predictive and prognostic value of several parameters. Furthermore, the limited number of patients who received endoscopic KSS made it necessary to pursue a multi-institutional approach. With the use of multiple imputation, assuming that the missing data were missing at random (MAR), we could reduce selection bias and improve the overall quality of data by including all identified patients irrespective of missing values. However, the MAR assumption is not devoid of irregularities, potentially introducing bias. The small number of patients contributed by several centers, reflecting both the rarity of UTUC managed with an endoscopic approach and the strict application of our inclusion and exclusion criteria, combined with the absence of a standardized management protocol across participating institutions, further limits the generalizability of our findings. We were unable to adjust for the clinical factors that drove the timing of second-look URS in individual patients. For example, an early re-look prompted by suspected residual disease versus routine scheduling. This may introduce confounding by indication and contribute to lead-time bias in the observed association between timing and RFS. Prospectively collected data on the reasons for timing are needed to account for this confounding. We considered a center-level random-effects model but did not pursue it, since patient numbers and treatment settings varied widely between centers and several centers contributed only a small number of patients each. Under these conditions, such a model would likely produce unstable estimates. We instead treat center-level heterogeneity, together with differences in treatment settings, devices, and follow-up protocols over our 13-year study period, as an inherent limitation of this multicenter retrospective design. Adjuvant intravesical or upper tract topical therapy was not standardized across participating centers, particularly during the study period (2004–2017), consistent with the broader lack of consensus on adjuvant endocavitary treatment for UTUC at that time [9]. Therefore, we could not account for its potential effect on recurrence or survival in our models. Tracking intravesical recurrence was not an aim of this study and was therefore not collected across participating centers; this is a limitation of the dataset. Diagnostic and treatment modality have been shown to influence subsequent intravesical recurrence risk in other UTUC cohorts [33], and this may be a useful direction for future analysis. Treatment decisions made after second-look URS, such as conversion to radical nephroureterectomy in patients with high-grade disease, were not an aim of this study and were therefore not captured. Indeed, this represents an important direction for future work. The relatively short median follow-up for the RFS analysis (12 months) limits our ability to draw conclusions about long-term recurrence. Moreover, the small number of cancer-specific deaths means the CSM findings should be interpreted as exploratory rather than definitive.

## 5. Conclusions

Early recurrence or residual disease at second-look URS appears to be the strongest prognostic factor for RFS and CSM in patients undergoing endoscopic KSS. Female gender and tumor size >1 cm are independent predictors for recurrence at second-look URS. Furthermore, our results suggest that second-look URS should be performed between 5 and 12 weeks after initial endoscopic treatment with an optimal timing of approximately 8 weeks. Given that this interval was derived and tested in the same cohort, this finding should be regarded as hypothesis-generating and requires external validation before being adopted as a clinical standard. The implementation of these factors into the decision-making process could help in adequate patient selection for endoscopic KSS, thereby potentially improving oncologic outcomes for UTUC patients.

## Figures and Tables

**Table 1:** Patients characteristics.

	Recurrence at Second-Look URS						
	No		Yes		Total		*p*-Value
	No.	%	No.	%	No.	%	
**Age** [median/IQR]	70	63–78	74	66–80	71	64–79	**0.035**
**Gender**							
Male	90	79.6	41	62.1	131	73.2	**0.011**
Female	23	20.4	25	37.9	48	26.8	
**Previous bladder cancer**							
Absent	66	58.4	44	66.7	110	61.5	0.3
Present	47	41.6	22	33.3	69	38.5	
**Tumor location**							
Pelvic-caliceal	60	53.1	39	59.1	99	55.3	0.3
Proximal ureter	8	7.1	4	6.1	12	6.7	
Middle ureter	11	9.7	3	4.5	14	7.8	
Distal ureter	29	25.7	13	19.7	42	23.5	
Both ^a^	5	4.4	7	10.6	12	6.7	
**Laterality**							
Left side	60	53.1	37	56.1	97	54.2	0.7
Right side	53	46.9	29	43.9	82	45.8	
**Multifocality**							
Absent	99	87.6	51	77.3	150	83.8	0.2
Present	13	11.5	14	21.2	27	15.1	
*Missing*	1	0.9	1	1.5	2	1.1	
**Hydronephrosis**							
Absent	87	77.0	54	81.8	141	78.8	0.1
Present	13	11.5	10	15.2	23	12.8	
*Missing*	13	11.5	2	3.0	15	8.4	
**Tumor size**							
≤1 cm	33	29.2	6	9.1	39	21.8	**0.015**
1.1–2 cm	31	27.4	20	30.3	51	28.5	
>2 cm	14	12.4	10	15.2	24	13.4	
*Missing*	35	31.0	30	45.5	65	36.3	
**Biopsy grading**							
Gx/low-grade	66	58.4	29	43.9	95	53.1	0.057
High-grade	14	12.4	6	9.1	20	11.2	
*Missing*	33	29.2	31	47.0	64	35.8	
**Weeks to SL** [median/IQR]	11	7–13	7	6–9	9	6–13	**<0.001**
**Total**	113	63.1	66	36.9	179	100.0	

^a^ Both indicates simultaneous involvement of the pelvicalyceal system and part of the ureter. URS: ureterorenoscopy; IQR: interquartile range; SL: second look.

**Table 2:** Logistic regression analyses for predicting second-look recurrence.

	MV			
	OR (95% CI)	*p*-Value	OR (95% CI)	*p*-Value
**Age**	1.04 (1.00–1.07)	**0.026**	1.02 (0.98–1.05)	0.3
**Female gender**	2.39 (1.21–4.69)	**0.012**	2.28 (1.11–4.70)	**0.026**
**Previous BC**	0.70 (0.37–1.32)	0.3		
**Tumor location**				
Pelvic-caliceal	Ref.			
Proximal ureter	0.78 (0.22–2.73)	0.7		
Medial ureter	0.42 (0.11–1.60)	0.2		
Distal ureter	0.69 (0.32–1.49)	0.3		
Both ^a^	2.15 (0.64–7.27)	0.2		
**Laterality**				
Left side	Ref.			
Right side	0.88 (0.48–1.62)	0.7		
**Multifocality**	1.88 (0.79–4.47)	0.2		
**Hydronephrosis**	1.26 (0.51–3.12)	0.6		
**Tumor size**				
≤1 cm	Ref.			
>1 cm	3.57 (1.33–9.61)	**0.012**	3.29 (1.15–9.45)	**0.027**
**HG biopsy**	1.09 (0.40–2.93)	0.9		

^a^ Both indicates simultaneous involvement of the pelvicalyceal system and part of the ureter. MV: multivariable analyses; OR: Odds ratio; CI: confidence interval; BC: bladder cancer; HG: high-grade.

**Table 3:** Cox proportional hazard regression analyses for RFS.

	UV		MV	
	HR (95% CI)	*p*-Value	HR (95% CI)	*p*-Value
**Age**	1.00 (0.98–1.02)	0.9		
**Female gender**	1.38 (0.87–2.20)	0.2		
**Previous BC**	0.81 (0.52–1.26)	0.3		
**Tumor location**				
Pelvic-caliceal	Ref.			
Proximal ureter	1.03 (0.44–2.41)	0.9		
Medial ureter	0.76 (0.30–1.90)	0.6		
Distal ureter	0.64 (0.36–1.12)	0.1		
Both ^a^	0.77 (0.35–1.72)	0.5		
**Laterality**				
Left side	Ref.			
Right side	1.04 (0.68–1.59)	0.2		
**Multifocality**	1.04 (0.60–1.81)	0.9		
**Hydronephrosis**	1.18 (0.64–2.15)	0.6		
**Tumor size**				
>1 cm	1.07 (0.58–1.96)	0.8		
>2 cm	1.27 (0.72–2.26)	0.4		
**HG biopsy**	0.71 (0.34–1.47)	0.4		
**SL recurrence**	3.94 (2.55–6.07)	**<0.001**	4.95 (3.07–7.98)	**<0.001**
**Weeks to SL**				
**≥13**	Ref.		Ref.	
**≤4**	1.25 (0.67–2.34)	0.5	0.73 (0.34–1.59)	0.4
**5–12**	0.96 (0.61–1.53)	0.9	0.54 (0.33–0.88)	**0.014**

^a^ Both, in terms of tumor location, means that the pelvic-caliceal system and some part of the ureter are affected; UV: univariable analyses; MV: multivariable analyses; HR: hazard ratio; CI: confidence interval; BC: bladder cancer; HG: high-grade; SL: second look.

**Table 4:** Cox proportional hazard regression analysis for CSM.

	UV	
	HR (95% CI)	*p*-Value
**Age**	1.00 (0.94–1.07)	0.9
**Female gender**	3.23 (0.98–10.7)	0.054
**Previous BC**	0.69 (0.21–2.31)	0.5
**Tumor location**		
Pelvic-caliceal	Ref.	
Proximal ureter	1.03 (0.16–10.8)	0.8
Medial ureter	-	1.0
Distal ureter	0.63 (0.13–3.05)	0.6
Both ^a^	1.78 (0.36–8.66)	0.5
**Laterality**		
Left side	Ref.	
Right side	0.68 (0.20–2.29)	0.5
**Multifocality**	1.51 (0.41–5.61)	0.5
**Hydronephrosis**	0.61 (0.08–4.59)	0.6
**Tumor size**		
>1 cm	0.45 (0.08–2.46)	0.4
>2 cm	1.39 (0.30–6.44)	0.7
**HG biopsy**	1.88 (0.47–7.59)	0.4
**SL recurrence**	5.10 (1.35–19.2)	**0.016**
**Weeks to SL**		
**≥13**	Ref.	
**≤4**	6.59 (1.08–40.3)	**0.041**
**5–12**	2.87 (0.59–13.9)	0.2

^a^ Both, in terms of tumor location, means that the pelvic-caliceal system and some part of the ureter are affected; UV: univariable analyses; HR: hazard ratio; CI: confidence interval; BC: bladder cancer; HG: high-grade; SL: second look.

## Data Availability

The data are not publicly available due to privacy and ethical restrictions, as they contain sensitive patient information collected under institutional data-sharing agreements between participating centers.

## Abbreviations

The following abbreviations are used in this manuscript:

UTUC	Upper tract urothelial carcinoma
UCB	Urothelial carcinoma of the bladder
RNU	Radical nephroureterectomy
KSS	Kidney-sparing surgeries
URS	Ureterorenoscopy
RFS	Recurrence-free survival
CSM	Cancer-specific mortality
CTU	Computed Tomography Urography
MAR	Missing at random

## Appendix A

**Table A1:** Imputed variables for logistic and Cox proportional hazard regression analyses.

Variable	Observations per m			
	Complete	Incomplete	Imputed	Total
Multifocality	177	2	2	179
Hydronephrosis	164	15	15	179
Tumor size	114	65	65	179
Biopsy grading	115	64	64	179

Complete + incomplete = total; imputed is the minimum across m of the number of filled-in observations.
